# Interaction of Coumarin Phytoestrogens with ER_α_ and ER_β_: A Molecular Dynamics Simulation Study

**DOI:** 10.3390/molecules25051165

**Published:** 2020-03-05

**Authors:** Ting Wang, Yunfei Wang, Xuming Zhuang, Feng Luan, Chunyan Zhao, M. Natália D. S. Cordeiro

**Affiliations:** 1 College of Chemistry and Chemical Engineering, Yantai University, Yantai 264005, China; 18865557652@163.com (T.W.); yunfeiw0712@163.com (Y.W.); xmzhuang@iccas.ac.cn (X.Z.); 2 School of Pharmacy Lanzhou University, Lanzhou 730000, China; zhaocy0225@hotmail.com; 3 LAQV/REQUIMTE, Department of Chemistry and Biochemistry, Faculty of Sciences, University of Porto, 4169-007 Porto, Portugal; ncordeir@fc.up.pt

**Keywords:** coumarin, phytoestrogens, estrogen receptor, molecular dynamics simulation, binding free energy

## Abstract

Coumarin phytoestrogens, as one of the important classes of phytoestrogens, have been proved to play an important role in various fields of human life. In this study, molecular simulation method including molecular docking and molecular dynamics methods were performed to explore the various effects between four classical coumarin phytoestrogens (coumestrol, 4-methoxycoumestrol, psoralen and isopsoralen), and estrogen receptors (ER_α,_ ER_β_), respectively. The calculated results not only proved that the four coumarin phytoestrogens have weaker affinity than 17β-estradiol to both ER_α,_ and ER_β_, but also pointed out that the selective affinity for ER_β_ is greater than ER_α_. In addition, the binding mode indicated that the formation of hydrogen bond and hydrophobic interaction have an important effect on the stability of the complexes. Further, the calculation and decomposition of binding free energy explored the main contribution interactions to the total free energy.

## 1. Introduction

Phytoestrogens are a class of non-steroidal compounds presence in plants that bind to estrogen receptors (ER) in mammals and humans [1,2]. For the chemical structures, most of the phytoestrogens with heterocyclic polyphenols are similar to estrogens (such as 17β-estradiol) in mammals. In life process, phytoestrogens and ER combine to have a dual regulation effect, which can exert both estrogen-like effects and anti-estrogen effects. Therefore, phytoestrogens play an important role in the prevention and treatment of menopausal symptoms, breast cancer, osteoporosis and cardiovascular diseases. Due to the special mechanism of phytoestrogens, they now have been used as a natural hormone replacement to treat the estrogen-related diseases [3,4,5].

So far, among the discovered phytoestrogens, they can be divided into several categories according to their chemical structures [6]: isoflavones, coumarins, lignans, stilbenes, triterpenoids, sterols and others [7,8,9]. Phytoestrogens are widely found in various types of food diet, such as grains, fruits, and vegetables. For instance, isoflavones are mainly found in Legumes; lignans are found in grains, such as flaxseed, rye, etc., and coumarins are mainly distributed in plants including Leguminosae, Compositae, Apiaceae and Rutaceae. As we known, there are many studies that have focused on the isoflavone phytoestrogens in various fields. Some researchers also described the use of isoflavone phytoestrogens in breast cancer, osteoporosis and cardiovascular diseases as well as practice in scientific research [10,11,12,13,14,15]. Similarly, coumarin phytoestrogens are another kind of important phytoestrogens, and their effects cannot be ignored. The basic structure of coumarin phytoestrogens is benzo-α-pyrone, such as coumestrol, psoralen, isopsoralen, osthole, imperatorin and the like [16,17].

The above-mentioned compounds have also been proved to have a preventive or therapeutic effect on some diseases. Wu et al. [18] showed that coumestrol exerts a chemotherapeutic effect on gynecological tumors through the PI3K and ERK1/2-MAPK pathways, and is a potential novel treatment that prevents against ovarian cancer development. Zhao et al. [19] found that psoralen has the estrogen-like effect through cell experiments, but its affinity for ER is weaker than that of estradiol. It is further found that psoralen has a significant up-regulation effect on the expression level of ER_α_ protein. Rajesh et al. [20] performed a 4D-QSAR study on coumarin derivatives and performed geometric optimization and molecular dynamics simulations for each compound. The results indicated that van der Waals interactions are important for increasing the activity of these compounds. For this, it is important to give further studies of the mechanism of coumarins at the molecular level.

**Figure 1:**
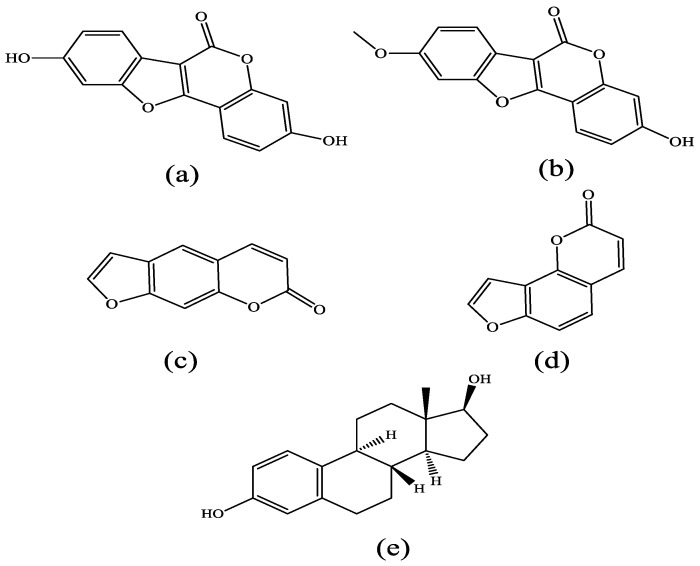
Chemical structure of four coumarin phytoestrogens: (**a**) coumestrol, (**b**) 4-methoxycoumestrol, (**c**) psoralen, (**d**) isopsoralen as well as (**e**) 17β-estradiol.

In this study, four typical coumarin phytoestrogens were selected for computational studies, namely coumestrol, 4-methoxycoumestrol, psoralen and isopsoralen (See Figure 1). For the estrogen-related compounds, they may have interaction or selection to the two estrogen receptor subtypes, ER_α_ and ER_β_. As is well known, the structures of ER_α_ and ER_β_ are similar, the amino acid residues therein differ, resulting in different effects of the two subtypes on the ligands.

Therefore, the four coumarin phytoestrogens were docked with the two subtypes of ER (ER_α_ and ER_β_) respectively. Then the complexes were explored by molecular dynamics simulation. The binding affinity of the ligands to the estrogen receptors and the interactions between the ligands and the receptors were investigated. In this study, 17β-estradiol, as a typical steroidal estrogen having a higher binding capacity than phytoestrogens [21,22] was used as a reference to compare with the selected coumarin phytoestrogens, and the differences between them indicate the strength of combining ability of the coumarin phytoestrogens.

## 2. Results and Discussion

### 2.1. Docking Analysis

In the present study, each of the four coumarin phytoestrogens acted as a ligand to bind to the estrogen receptor (ER_α_ and ER_β_). The docking results (docking scores) were listed in Table 1. The smaller the docking score, the more stable the complex formed. The results of docking showed that the affinity of coumarin phytoestrogens for ER_α_ and ER_β_ is generally lower than 17β-estradiol for both ER_α_ and ER_β_ [23,24]. From the docking results of coumestrol, it can be seen that the docking score of coumestrol with ER_β_ is significantly higher than that of ER_α_, which proves that coumestrol has a stronger affinity for ER_β_ than ER_α_. This result is consistent with the results of Zafar et al. [25]. For the corresponding system of ER_α_ and ER_β_, the affinity of coumestrol and 4-methoxycoumestrol is higher than that of psoralen and isopsoralen. The results of the binding affinity of isopsoralen and psoralen for ER_α_ are consistent with the results of the reference [26], that is, the docking score of isopsoralen is slightly lower than that of psoralen. In this study, the docking results do not make a major conclusion, but served as a reference for the subsequent research.

### 2.2. Molecular Dynamics Simulation Analysis

**Figure 2:**
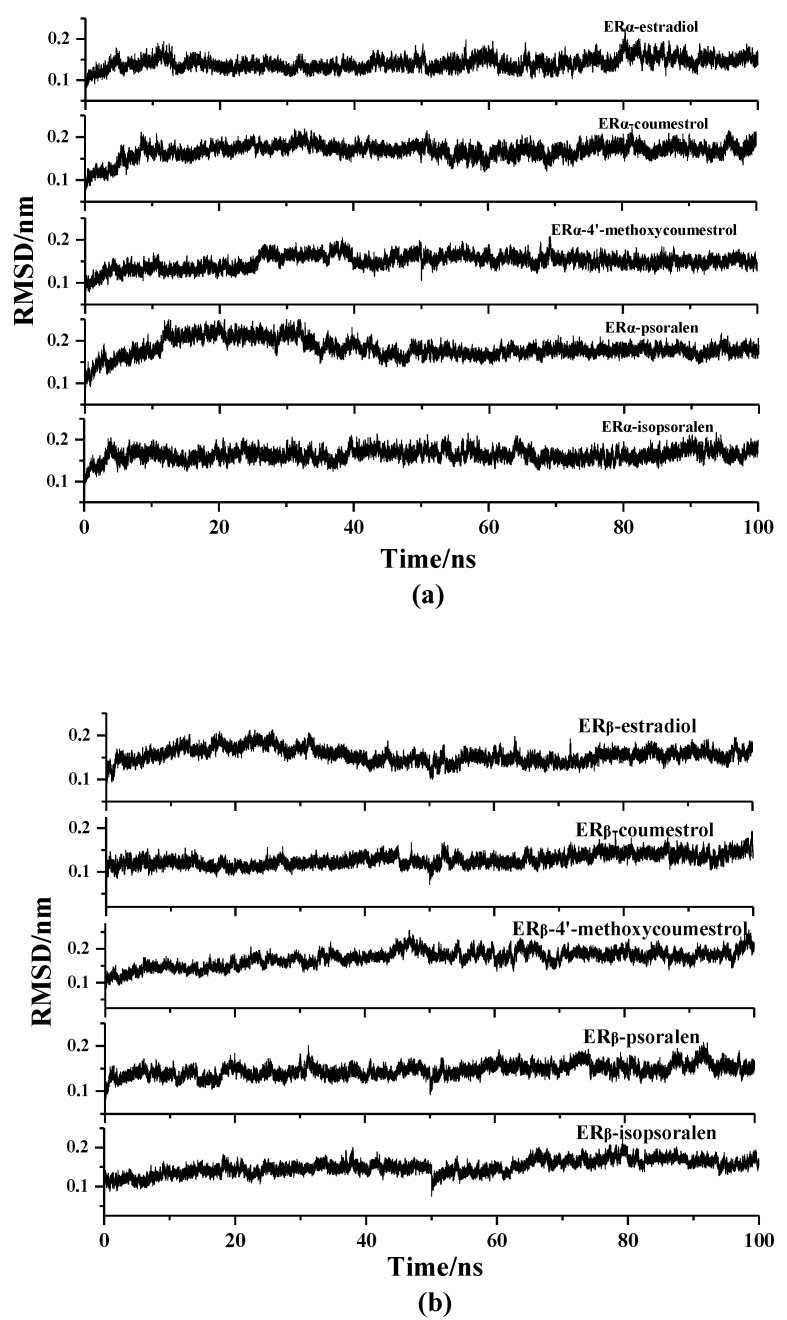
RMSD values of ER-ligand complexes in 100 ns MD simulation (**a**) in the ER_α_ system, (**b**) in the ER_β_ system.

In order to evaluate the structural stability of the complexes during the simulation process, the Root Mean Square Deviation (RMSD) values of the ER_α_-ligands system and ER_β_-ligands system were separately analyzed using the Ptraj program in Amber 14. Figure 2 shows the RMSD values of the complexes in the ER_α_-ligands (a) and ER_β_-ligands (b) system. It can be seen from the Figure that the conformation of all the complexes reached a steady state after simulation of 100 ns. In the ER_α_ system (Figure 2a), the mean of the RMSD of the stable conformations fluctuates around 0.16 nm, and the mean of the RMSD of the ER_β_ system fluctuates around 0.14 nm (Figure 2b), indicating that the ER_β_ system has less fluctuation than the ER_α_ system during the MD simulation.

**Figure 3:**
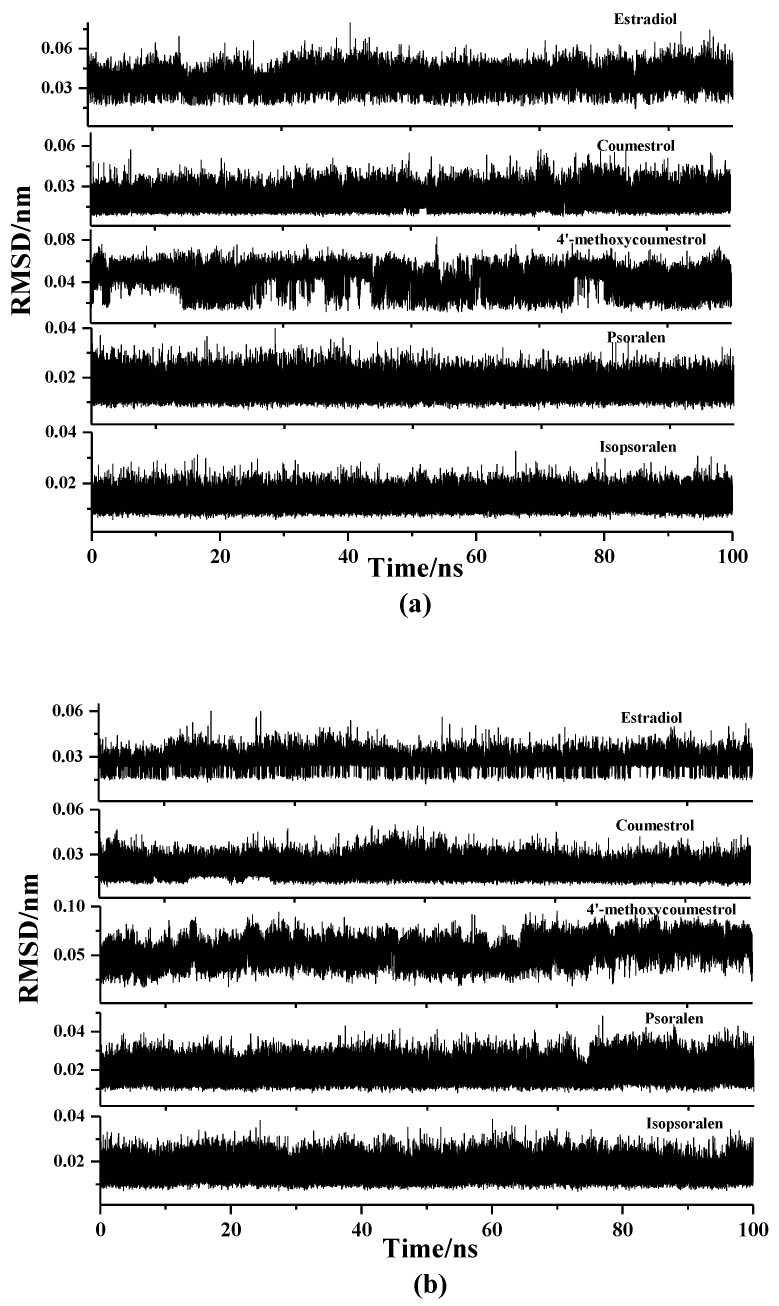
RMSDvalues of the four coumarin phytoestrogens as well as 17β-estradiol (**a**) in the ER_α_ system, (**b**) in the ER_β_ system.

Similarly, in order to evaluate the changes of the ligands themselves during the MD simulation, the RMSD values of the ligands were also calculated, which were shown in Figure 3. The results showed that the RMSD values of all the ligands fluctuate within a small range, that is, the values were all below 0.1 nm, indicating that the ligands were in a stable state during the simulation process, and contribute positively to the formation of the complexes.

**Figure 4:**
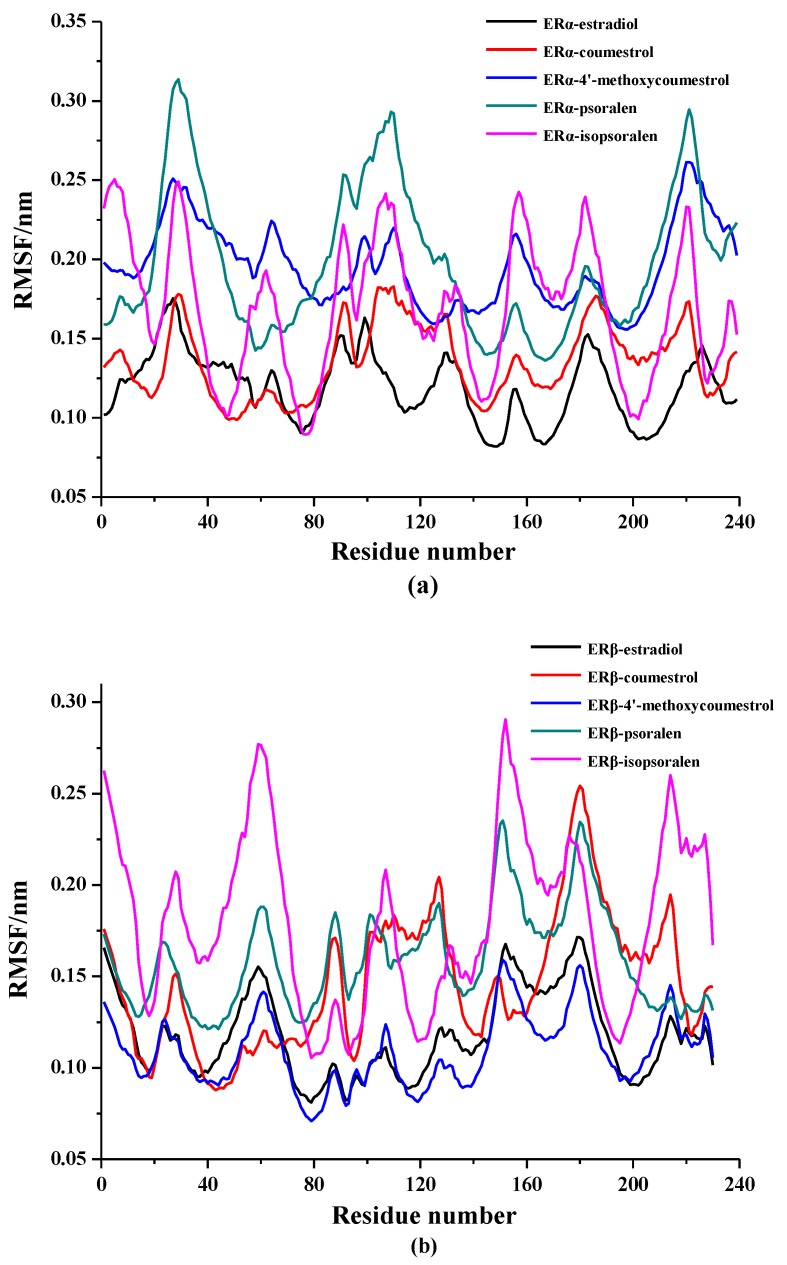
RMSF values of ER-ligand complexes in 100 ns MD simulation (**a**) in the ER_α_ system, (**b**) in the ER_β_ system.

The root means square fluctuation (RMSF) values can be calculated to evaluate the stability of each amino acid in the complexes throughout the simulation. The results are shown in Figure 4. According to the curve trend in the figure, it can be seen that the fluctuation trend of the complexes in the same system are similar to the trend of the complex of ER-estradiol, indicating that the formation process of all the complexes are similar. Among them, the RMSF curve of the complexes containing coumestrol and 4-methoxycoumestrol showed even less fluctuation, compared to the RMSF curve of the complexes containing psoralen and isopsoralen, which are consistent with the docking results. The complex ER_α_-estradiol has lower RMSF values around PRO20, GLU45, LEU76, LEU120, LEU145, ARG166, HIE213 and GLU231. The other complexes of the four coumarin phytoestrogens in the ER_α_ system also have lower RMSF values at these similar positions, indicating that these amino acids play a positive role in the stability of the complexes. The amino acids in the ER_β_ system are ARG21, LEU38, MET78, ILE110, LEU142, VAL165, HIE207, and LEU223, which also performed the above-mentioned functions.

### 2.3. Binding Patterns Analysis

**Figure 5:**
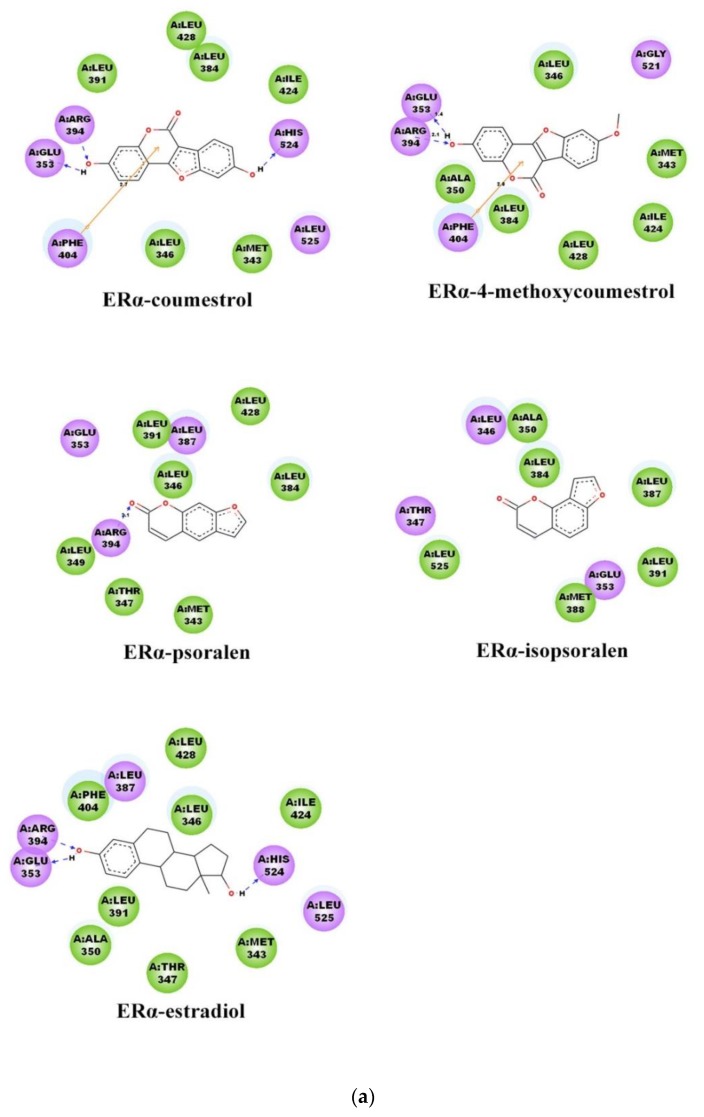

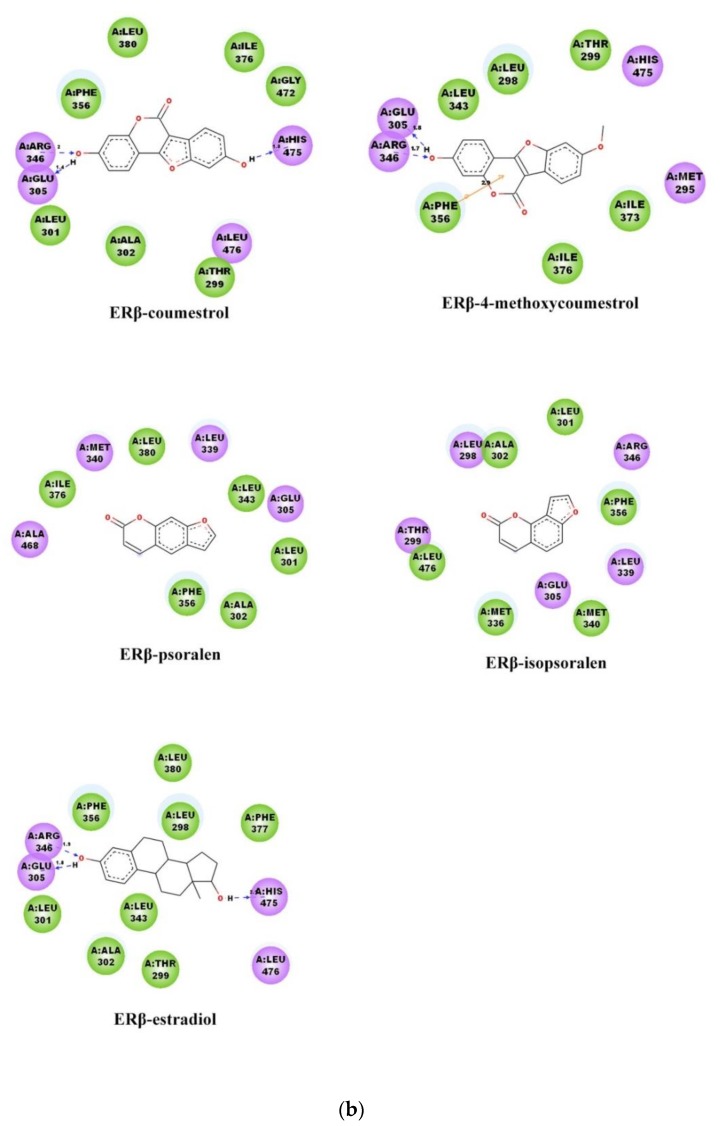
Interaction between ligands and proteins: (**a**) in the ER_α_ system; (**b**) in the ER_β_ system.

Figure 5 shows the interaction of proteins with ligands. In the figure, the arrow indicates a hydrogen bond formed between ligands and amino acids, the arrow points to the donor, and the value between hydrogen bonds indicates the bond length. The results showed that 17β-estradiol forms three hydrogen bonds with the two receptors ER_α_, and ER_β_, respectively. The hydrogen bond definition standard for this process is that the distance between the donor and the acceptor is less than 0.35 nm, and the bond angle is greater than 120°. The coumestrol formed three hydrogen bonds with the ER_α_, and ER_β_, respectively; while the 4-methoxycoumestrol formed two hydrogen bonds with them. Psoralen only formed one hydrogen bond with ER_α_, and no hydrogen bond was formed with ER_β_. Isopsoralen did not form any hydrogen bonds with either ER_α_ or ER_β_. According to the above results, the number of hydrogen bonds formed between coumestrol and ERs is the highest, followed by 4-methoxycoumestrol, psoralen and finally isopsoralen. Among them, the phenolic hydroxyl group of coumestrol forms hydrogen bonds with the residues at the two ends of the binding cavity, and the hydrogen bond interactions are greater than other ligands, which helps to improve the binding stability of coumestrol to ER. According to the references [27,28,29,30] and the comparison of the docking results, it can be seen that the hydrogen bond interactions play an important stabilizing role in the ligand-receptor binding process.

**Figure 6:**
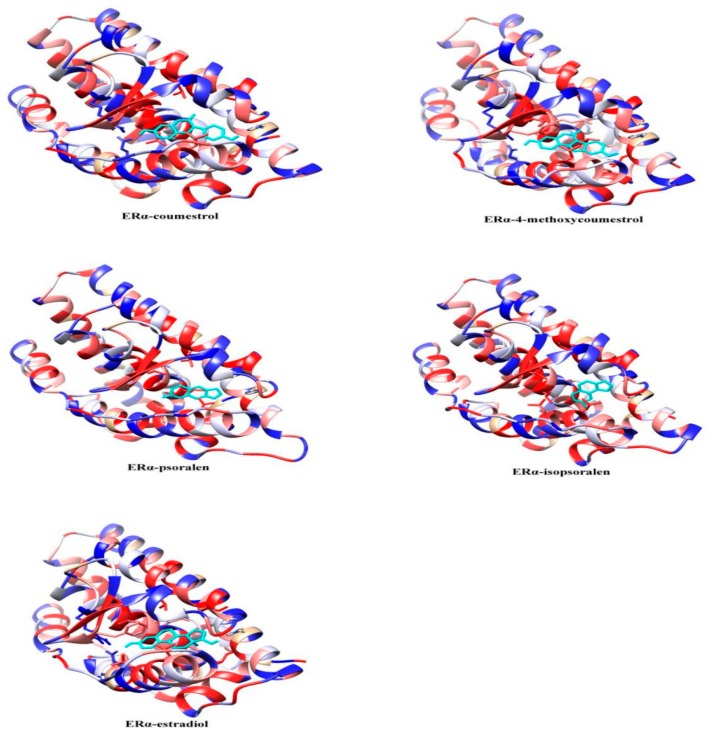

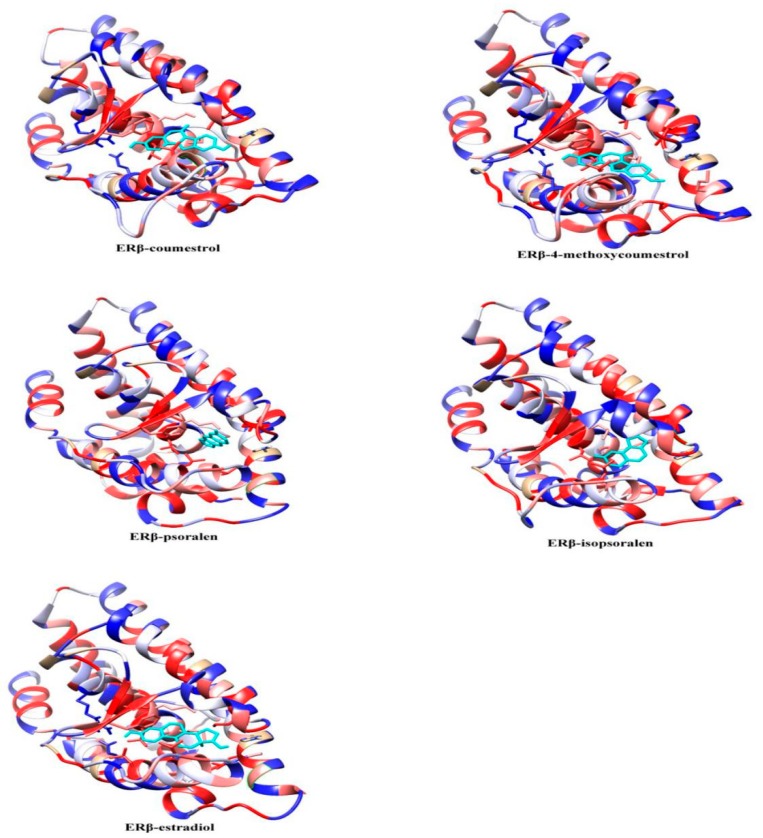
Hydrophobic interaction of complexes, the color from red to blue indicates a decrease in hydrophobic potential.

To further study the binding pattern between the ligand and the receptor, the hydrophobic interaction analysis of the complexes in the last 1ns of the MD simulation was performed using Chimera1.13 software (National Institutes of Health: Bethesda, MD, USA). The results are shown in Figure 6. The amino acid residues at the pocket of the ligand-protein binding are mostly hydrophobic in nature, creating hydrophobic interactions with receptors, and further maintaining the stability of the complexes. It can be seen from the figure that all ligands are in contact with the receptor proteins, indicating the interaction between them. The hydrophobic interactions of the complexes in Figure 6 are similar, that is, the hydrophobic amino acids at the left and bottom of the binding cavity are greater than other locations. This result is consistent with the reference [29], that hydrophobic interactions are the dominant force for stabilizing the binding of ligands to receptors.

### 2.4. Binding Free Energy Analysis

The calculation results of the binding free energy of the ER_α_-ligands and ER_β_-ligands system were shown in Table 2 and Table 3. By comparing the calculation results of the two systems, it can be seen that the binding free energy values of the complexes in the ER_β_ system are generally lower than the binding free energy of the ER_α_ system. The lower the binding free energy, the more stable the complexes formed, thus, implying that the complexes formed in the ER_β_ system are more stable. Furthermore, it is confirmed that the phytoestrogens have greater affinity for ER_β_ than ER_α_ [23,24]. In the ER_α_ system, according to the data in Table 2, the binding free energy value of the complex ER_α_-estradiol is the lowest, indicating that the complex is the most stable. This result is consistent with the above conclusions that estradiol has a higher affinity for ER than phytoestrogens. For the complexes ER_α_-4-methoxycoumestrol and ER_α_-coumestrol, their binding free energy values are higher than ER_α_-estradiol. The structure of 4-methoxycoumestrol and coumestrol differ only in the substituents on the benzene ring, that is, one is a methoxy and the other is a hydroxyl. The free energy value of the complex ER_α_-4-methoxycoumestrol is slightly lower than that of ER_α_-coumestrol, indicating that the structure of the ligands may also have a certain effect on the stability of the complexes. The free energy values of the other two complexes, ER_α_-psoralen and ER_α_-isopsoralen are similar and somewhat higher, indicating that their stability is not as good as the former two. The complexes in the ER_β_ system have the same trend as the ER_α_ system, indicating that among the four phytoestrogens, 4-methoxycoumestrol has the highest affinity for both ER_α_ and ER_β_, followed by coumestrol, psoralen and finally isopsoralen.

According to the energy contribution data of van der Waals energy, electrostatic interaction, polar solvation energy and non-polar solvation energy, one can see that the van der Waals energy has the largest contribution (the energy value is the smallest). In addition, during the formation of the complexes, the van der Waals energy, electrostatic interaction energy and non-polar solvation energy in the ER_α_-ligands and ER_β_-ligands systems are negative, indicating that these three effects are beneficial to the combination of ligands and acceptors; the value of the polar solvation energy is positive, indicating that it hinders the formation of the complexes.

In order to compare our results with the experimental results, we searched the clinical assays reported in ChEMBL (https://www.ebi.ac.uk/chembl/) for these compounds. However, there are only two compounds that can be found, that is, the IC_50_ values of ER_α_-coumestrol and ER_α_-estradiol as well as ER_β_-estradiol. The results are shown in Table 4. As can be seen in Table 4, the IC_50_ values of estradiol binding to ER_α_ and ER_β_ are both 2 nM, which are lower than the IC_50_ values of other complexes, indicating that the affinity of estradiol is higher than that of coumarin phytoestrogens. In addition, the IC_50_ value of ER_α_-coumestrol is higher than ER_β_-coumestrol, which is also consistent with our calculation results of binding energy.

### 2.5. Binding Free Energy Decomposition

**Figure 7:**
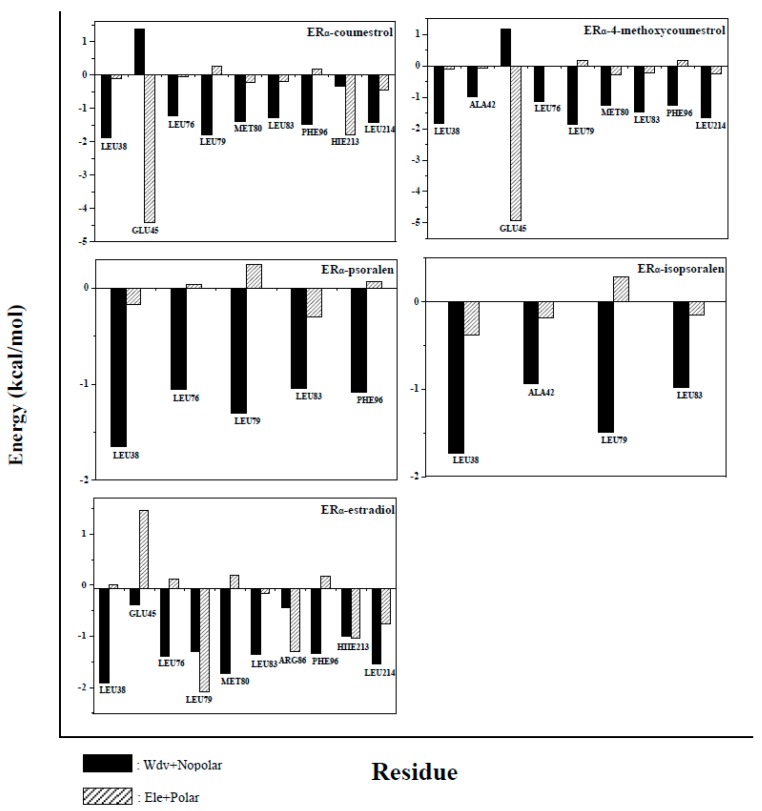
Contribution of key amino acids to binding free energy in the ER_α_ system. Black part: van der Waals and non-polar solvation energy; gray part: electrostatic energy and polar solvation energy. The positive values in the Figure are not conducive to the binding of the ligand to the receptor, and the negative value facilitates the binding of the ligand to the receptor.

**Figure 8:**
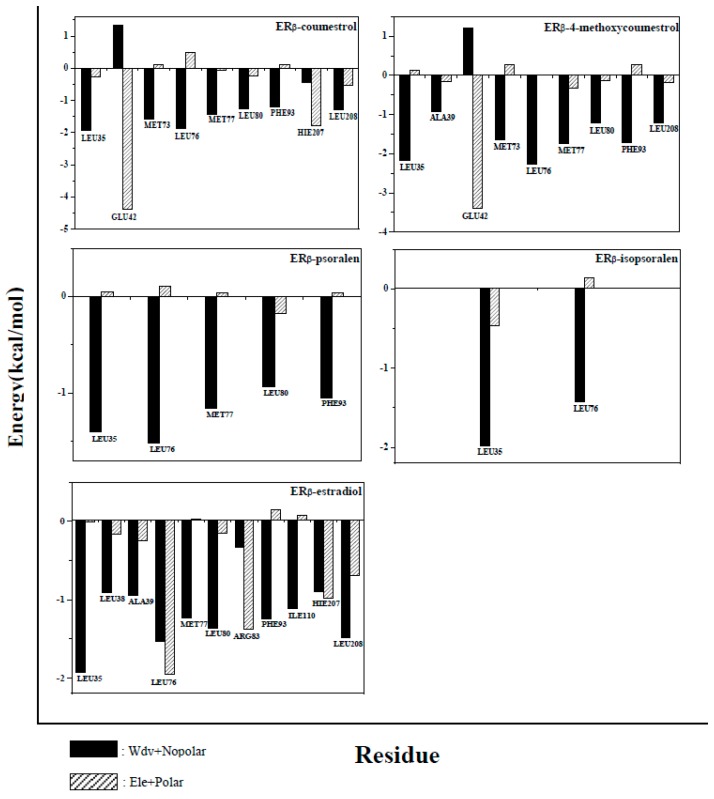
Contribution of key amino acids to binding free energy in the ER_β_ system. Black part: van der Waals and non-polar solvation energy; gray part: electrostatic energy and polar solvation energy. The positive values in the figure are not conducive to the binding of the ligand to the receptor, and the negative value facilitates the binding of the ligand to the receptor.

All amino acids of the protein were analyzed, in order to further understand the contribution of each amino acid to the binding free energy during complexes formation. Amino acids that contribute greatly to the binding free energy (more than −1 kcal/mol) were defined as key amino acids, and energy analysis was performed on these key amino acids. The results of the analysis were shown in Figure 7 and Figure 8. In the ER_α_ system, the number of key amino acids in the complexes is 9, 9, 5, 4, and 10, respectively (Residues name and sequence were shown in Figure 7). The number of key amino acids in the ER_β_ system is 9, 9, 5, 2, and 10 (Residues name and sequence were shown in Figure 8). The larger the number of key amino acids, the more stable the complexes formed. The above results indicate that the complexes ER_α_-estradiol and ER_β_-estradiol are the most stable of all complexes (The two compounds have the largest number of key amino acids, 10). The number of key amino acids in complexes ER_α_-4-methoxycoumestrol (9), ER_β_-4-methoxycoumestrol (9), ER_α_-coumestrol (9) and ER_β_-coumestrol (9) are same and greater than the number of key amino acids in the complexes ER_α_-psoralen (5), ER_β_-psoralen (5), ER_α_-isopsoralen (4), and ER_β_-isopsoralen (2), which imply that the former are more stable than the latter. This is consistent with the above analysis. Almost all of these key amino acids are located around the binding cavity of the ligands and the receptor proteins, and the fluctuation is small during the MD simulation process, and the contribution to the binding free energy is large.

In the energy analysis process of the key amino acids, van der Waals energy and non-polar solvation energy were defined as hydrophobic interactions (black part in the figure), and electrostatic energy and polar solvation energy were defined as electrostatic interactions (grey part in the Figure). According to the contribution of key amino acids in Figure 7 and Figure 8, the hydrophobic interactions are mostly to favor the formation of complexes (the energy is negative), while the electrostatic interactions are more likely to be detrimental to the formation of complexes (the energy is positive). In summary, the hydrophobic interactions promote the binding of ligands to receptors.

## 3. Materials and Methods

### 3.1. Protein and Ligand Preparation

The initial structures of the estrogen receptors ER_α_ (PDB ID: 1GWR) and ER_β_ (PDB ID: 3OLS) were obtained from the RCSB Protein Data Bank [33]. The ligand binding domain of ER_α_ and ER_β_ were extracted from the initial structures for the subsequent studies. Preparation of the receptor proteins (ER_α_ and ER_β_) were performed by the Protein Preparation Wizard module of the Schrodinger 2015-2 software (Schrodinger, Inc., NY, USA) [34]. The process of preparations included the removal of water molecules, the addition of hydrogen atoms, and the optimization of the conformations under the Schrodinger’s OPLS_2005 force field [35].

The structure files in SDF format for all the ligands were obtained from the Pubchem database [36]. The ligands were ionized and optimized using the Ligprep Wizard module of the Schrodinger software (Schrodinger, Inc., NY, USA) [37], and the preparation process considered all possible conformations. Finally, the most suitable ligand conformation was selected for molecular docking.

### 3.2. Molecular Docking Studies

First, a small molecule ligand was used as a center to prepare a 3D space grid with the residues surrounding the binding site. Then, the prepared ligands and the receptor proteins were docked using the Glide 6.7 docking procedure in Schrodinger 2015-2 (Schrodinger, Inc., NY, USA) [38,39,40]. Due to the existence of certain deviations in various docking methods, the docking results were only used for reference and did not make the key conclusions. Finally, the complexes formed by docking were used for molecular dynamics simulation studies.

### 3.3. Molecular Dynamics Simulations

The molecular dynamics (MD) simulation process was performed using the Amber14 program (University of California: San Francisco, CA, USA) [41,42]. The parameters of the system were generated by Antechamber module in Amber program. The restrained electrostatic potential (RESP) was used to describe the partial atomic charges. The FF03 force field [43] in Amber was used to describe the molecular parameters of the protein, and the molecular parameters of the ligand were described using the GAFF force field [44]. The complexes were hydro-treated using the tleap module. The complexes were dissolved in a TIP3P water molecule box with a distance of at least 12Å from the edge of the water box. For the ER_α_ system, five sodium cations were added to neutralize the negative charge [45], making the system electrically neutral. Two sodium cations were also added to the ER_β_ system for the same purpose.

The purpose of energy minimization was to make the system more balanced. In the optimization process of each step, the steepest descent method of 3000 steps was firstly performed, and then the conjugate gradient method of 2000 steps was optimized. The first optimization was performed to minimize the solvent; then to constrain the counter ions for energy minimization. Finally, the entire system was minimized without restrictions. The interception distance of a long-range Coulombic interaction was set to 1 nm using the Particle Mesh Ewald (PME) method [46]. The SHAKE algorithm was used to limit the key length, and the integration step in the MD simulation process was set to 2 fs. Subsequently, the temperature rise dynamics of the system was run. The temperature was raised from 0 K to 300 K in 50 ps, and the collision frequency was 2 ps^−1^. Then, the system was subjected to an unrestricted equilibrium dynamics of 500 ps. Finally, all systems continue to run for 100 ns in a normal temperature, constant pressure NPT system, and the trajectory calculation interval was 1 ps.

The Root Mean Square Deviation (RMSD) and Root Mean Square Fluctuation (RMSF) were used to describe the structural features, which were calculated by the Ptraj module [47] of Amber14 (University of California: San Francisco, CA, USA).

### 3.4. Binding Free Energy Calculations and Energy Decomposition

The calculation of the binding free energy of all complexes was obtained by the MMPBSA.py script [48] in Amber14 software (University of California: San Francisco, CA, USA). This process extracted a snapshot from the trajectory file after the MD simulation and then performed the calculation. The binding free energy of the complexes was calculated as follows:

(1) $$\Delta G_{\mathrm{bind}}=\Delta G_{\mathrm{comp}}-\Delta G_{\mathrm{pro}}-\Delta G_{\mathrm{lig}}=\Delta G_{\mathrm{MM}}+\Delta G_{\mathrm{sol}}-T\Delta S$$

(2) $$\Delta G_{\mathrm{MM}}=\Delta G_{\mathrm{int}}+\Delta G_{\mathrm{ele}}+\Delta G_{\mathrm{vdw}}$$

(3) $$\Delta G_{\mathrm{solv}}=\Delta G_{\mathrm{pol}}+\Delta G_{\mathrm{nopol}}$$

Among them, the binding free energy (ΔG_bind_) was composed of the binding free energy of the complex (ΔG_comp_), the receptor protein (ΔG_pro_) and the ligand (ΔG_lig_). The gas phase binding energy (ΔG_MM_) was composed of internal energy (ΔG_int_), electrostatic interaction energy (ΔG_ele_) and van der Waals energy (ΔG_vdw_); the solvation free energy (ΔG_solv_) was calculated from the polar (ΔG_pol_) and non-polar (ΔG_nopol_) solvation free energy. For entropy change (TΔS), the change of entropy causes by conformational change after the ligand binds to the receptor. The purpose of this paper is only to compare the binding energy between several systems, and the calculation process of entropy change is more complicated, so the contribution of entropy change to binding free energy is neglected.

To form a complex by the ligand binding to the protein, each amino acid in the system contributed differently to the binding free energy. For the purpose of this analysis, the free energy decomposition process was used by the MM/GBSA free energy decomposition module in Amber14 software (University of California: San Francisco, CA, USA) to decompose the free energy and then explore the contribution of each amino acid. Among them, the contribution of three kinds of energy was investigated, that is, the van der Waals energy, the electrostatic interaction energy as well as the solvation energy.

## 4. Conclusions

In this paper, four coumarin phytoestrogens were used as ligands to dock with ER_α_ and ER_β_, and the binding mode along with the binding free energy between ligands and receptors were explored by molecular dynamics simulation, respectively. The results showed that the affinity of the complex 4-methoxycoumestrol was slightly stronger than that of coumestrol, and was significantly higher than that of psoralen and isopsoralen. The four coumarin phytoestrogens had greater affinity and selectivity to ER_β_ than ER_α_. In addition, the calculation and decomposition of the binding free energy pointed out that van der Waals energy and non-polar solvation energy play a beneficial role in the formation of complexes. In general, we hope that the results of this study can provide some help for further exploration of the weak interaction between the phytoestrogens and estrogen receptors.

## Figures and Tables

**Table 1:** Docking results of the complexes in the ER_α_ and ER_β_ system.

	ER_α_ System Docking Score (kcal/mol)	ER_β_ System Docking Score (kcal/mol)
Coumestrol	−9.805	−10.489
4-methoxycoumestrol	−8.677	−8.812
Psoralen	−7.107	−7.339
Isopsoralen	−7.311	−7.502
17β-estradiol	−10.784	−10.943

**Table 2:** Binding free energy of complexes in ER_α_ system.

Energy (kcal/mol)	ER_α_-Estradiol	ER_α_-Coumestrol	ER_α_-4-Methoxycoumestrol	ER_α_-Psoralen	ER_α_-Isopsoralen
ΔE_VDW_	−41.3422	−34.9511	−34.9963	−25.1273	−25.3492
ΔE_ELE_	−8.8830	−31.1511	−26.4485	−8.6063	−4.4464
ΔE_POL_	10.1237	37.4083	29.6470	16.1202	12.0756
ΔE_NPOL_	−5.2950	−4.8390	−5.2148	−3.4632	−3.4415
ΔG_GAS_	−50.2298	−66.1022	−61.4449	−33.7336	−29.7956
ΔG_SOLV_	4.8287	32.5693	24.4321	12.6569	8.6341
ΔG_TOT_	−45.4011	−33.5329	−37.0128	−21.0766	−21.1615

ΔE_VDW_ = val der waals energy; ΔE_ELE_ = electrostatic energy; ΔE_POL_ = polar solvent energy; ΔE_NPOL_ = nonpolar solvent energy; ΔG_GAS_ = ΔE_VDW_ + ΔE_ELE_; ΔG_SOLV_ = ΔE_POL_ + ΔE_NPOL_; ΔG_TOT_ = total binding free energy.

**Table 3:** Binding free energy of complexes in ER_β_ system.

Energy (kcal/mol)	ER_β_-Estradiol	ER_β_-Coumestrol	ER_β_-4-Methoxycoumestrol	ER_β_-Psoralen	ER_β_-Isopsoralen
ΔE_VDW_	−41.6851	−34.4483	−36.8087	−26.9760	−27.5848
ΔE_ELE_	−12.8699	−32.3476	−33.5825	−4.8503	−3.2704
ΔE_POL_	12.8858	35.7288	35.7121	11.8430	11.2447
ΔE_NPOL_	−5.1785	−5.0680	−5.2660	−3.6656	−3.6354
ΔG_GAS_	−54.5584	−66.7959	−70.3912	−31.8263	−30.8552
ΔG_SOLV_	7.7074	30.6607	30.4461	8.1774	7.6093
ΔG_TOT_	−46.8510	−36.1352	−39.9451	−23.6490	−23.2459

ΔE_VDW_ = val der waals energy; ΔE_ELE_ = electrostatic energy; ΔE_POL_ = polar solvent energy; ΔE_NPOL_ = nonpolar solvent energy; ΔG_GAS_ = ΔE_VDW_ + ΔE_ELE_; ΔG_SOLV_ = ΔE_POL_ + ΔE_NPOL_; ΔG_TOT_ = total binding free energy.

**Table 4:** Comparison of the results with the obtained physical measurements.

Complexes	Standard Type	Standard Value	Document Report
ER_α_-coumestrol	IC_50_	75.7 nM	Ref [31]
ER_α_-4-methoxycoumestrol	NA	NA	NA
ER_α_-psoralen	NA	NA	NA
ER_α_-isopsoralen	NA	NA	NA
ER_α_-estradiol	IC_50_	2 nM	Ref [32]
ER_β_-coumestrol	IC_50_	18.6 nM	Ref [31]
ER_β_-4-methoxycoumestrol	NA	NA	NA
ER_β_-psoralen	NA	NA	NA
ER_β_-isopsoralen	NA	NA	NA
ER_β_- estradiol	IC_50_	2 nM	Ref [32]

NA: not available.
